# Clustering of South Korean Adolescents’ Health-Related Behaviors by Gender: Using a Latent Class Analysis

**DOI:** 10.3390/ijerph18063129

**Published:** 2021-03-18

**Authors:** Myungah Chae, Sophia Jihey Chung

**Affiliations:** Red Cross College of Nursing, Chung-Ang University, Seoul 06974, Korea; chae0326@naver.com

**Keywords:** adolescents, gender, health-related behaviors, latent class analysis

## Abstract

Background: Health-related behaviors during adolescence could influence adolescents’ health outcomes, leading to either advantageous or deteriorative conditions. Clustering of adolescents’ health-related behaviors by gender identifies the target groups for intervention and informs the strategies to be implemented for behavioral changes. Methods: Data from 1807 adolescents in grades 7 and 10 in a city in South Korea were used. Health-related behaviors including eating habits, physical activity, hand washing, brushing teeth, drinking alcohol, smoking, and Internet use were examined. Latent class analysis (LCA) was used to identify subgroups of adolescents with regard to their health-related behaviors. Results: A four-class model was the most adequate grouping classification across genders: adolescents with (1) healthy behaviors, (2) neither health-promoting nor health-risk behaviors, (3) good hygiene behaviors, and (4) unhealthy behaviors. The majority of both male and female adolescents were classified into the healthy group. Male adolescents belonging to the healthy group were more likely to engage in vigorous physical activities, while vigorous physical activity was not important for female adolescents. The smallest group was the unhealthy group, regardless of gender; however, the proportion of boys in the unhealthy group was almost twice that of girls. Only female adolescents engaged in excessive Internet use, especially the group with neither health-promoting nor health-risk behaviors. Conclusion: To improve adolescents’ health-related behaviors, it would be more effective to develop tailored interventions considering the behavioral profiles of the target groups.

## 1. Introduction

Adolescence is the transitional period from childhood to adulthood. As adolescents become more independent from their parents, they establish their preferences and further develop their behaviors [1,2]. Behaviors established during adolescence, including health-related behaviors, could persist into adulthood and have an impact on their future health. Health-related behaviors such as physical activity, diet, alcohol consumption, and smoking could influence not only their present health status but also the rest of their lives [3,4,5,6,7]. Thus, comprehensively investigating adolescents’ health-related behaviors is crucial in order to identify how to best intervene and improve adolescents’ health.

Many studies have focused on adolescents’ health-related behaviors. However, there are differences with regard to gender in health-related behaviors. In general, male adolescents are more likely to engage in physical activities than female adolescents [8,9]. Female adolescents may be more concerned with their dietary behaviors, because they pay more attention to their body image or body shape [10,11,12]. Female adolescents are known to drink less alcohol than male adolescents [13,14]. They are also less likely to use tobacco products [13,15], although the prevalence of female adolescents’ light smoking has been increasing [16]. Therefore, gender differences should be considered in the development of interventions to improve the health-related behaviors of adolescents, which could lead to more effective results in the modification of behaviors.

Clustering of adolescents’ health-related behaviors could also be helpful for developing programs for adolescents. Certain health-related behaviors tend to occur in tandem. Previous studies found that alcohol use and smoking commonly co-occurred [17]. Additionally, studies have investigated the link between unhealthy dietary behaviors, low physical activity, and sedentary behavior [18,19,20]. As previously mentioned, the patterning of health-related behaviors during adolescence could become habitual, having a positive or negative impact on current and future health. By identifying the profile of adolescents’ health-related behaviors and thereby the health-risk groups, we could determine which groups and behaviors to target for intervention.

Latent class analysis (LCA) could be used to cluster adolescents’ health-related behaviors. LCA is a person-centered approach to classify smaller homogeneous clusters based on the probabilities of health-related behaviors reported by adolescents. This is a descriptive method for grouping individuals [21]. Some studies related to adolescents’ health-related behaviors, using LCA, have been done in Western cultures. A study examining health lifestyles among adolescents in the United States identified four classes [22]. The largest group was characterized by moderate exercise, unhealthy eating habits, and excessive computer use, while the smallest group had a lower probability of health-promoting behaviors. However, a study conducted in Australia identified three classes among adolescents: the moderate-risk group; the inactive, non-smokers group; and the smokers and binge-drinkers group. The moderate-risk group was the largest group and had the lowest probability of health-risk behaviors [23]. Olson and her colleagues [24] used a nationwide survey of adolescents in the United States in 1994 and 2008 and used clustering to compare gender differences in health behaviors. Although the three-class model was the most adequate model in both genders, the largest and the smallest clusters differed by gender. For female adolescents, the largest group was a mixed cluster that included moderate cigarette smoking, no physical activity, and higher consumption of fast foods, while for male adolescents, the largest group was the unhealthy group. The unhealthy group was the smallest group among female adolescents; however, for male adolescents, the smallest group was the healthy group that included a lower proportion of cigarette smoking (and other tobacco products) and binge drinking.

Depending on the culture and the time when the research is conducted, grouping of adolescents’ health-related behaviors could differ. In addition, gender could be an essential factor for the patterning of adolescents’ behaviors. However, few studies have examined the clustering of health-related behaviors among adolescents, especially in Eastern cultures. A study conducted in an Eastern cultural context would contribute to our understanding of adolescents’ patterns of health-related behaviors across cultures. Therefore, the aim of this study was to examine gender differences in the grouping of adolescents’ health-related behaviors in South Korea using LCA.

## 2. Material and Methods

### 2.1. Study Participants

Participants were students who participated in the 2016 Health Examination in School, which is conducted according to the School Health Law in South Korea. Physical and psychological health as well as medical assessment are included in this annual examination. Adolescents are asked to visit specific clinics for medical assessment and to answer questions on medical history, physical competence, and lifestyle (eating habits, physical activity, drinking and smoking habits). In addition, a survey questionnaire is completed in the home to assess psychological well-being (anxiety and depression). All 7th (aged from 12 to 13) and 10th grade adolescents (aged from 15 to 16) registered in all the schools in Seogwipo city of Jeju-island, South Korea, were included. After excluding incomplete questionnaires, 1807 adolescents were included in this secondary data analysis. Approval of the study was obtained from Chung-Ang University Institutional Review Board (IRB No. 2019-044).

### 2.2. Health-Related Behaviors

Health-related behaviors included eating habits, physical activity, personal hygiene, drinking and smoking behaviors, and Internet use. All responses to the questions were dichotomous, to be answered with “yes” or “no.” For eating habits, adolescents were asked whether they ate breakfast regularly, ate fruits and vegetables every day, ate fast food every day, and consumed milk or other dairy products every day. Adolescents were also asked whether they engaged in vigorous physical activity three times or more per week. Personal hygiene was assessed by asking whether they brushed their teeth more than twice a day and washed their hands before eating food or after going out. For drinking and smoking behaviors, they were asked whether they had any experience of drinking alcohol or smoking in the past 30 days. For excessive Internet use, adolescents were asked whether they used the Internet, including playing Internet games, for two hours or more per day.

### 2.3. Statistical Analysis

To determine the health-related behavior patterns, latent class analysis (LCA) was applied using Mplus, version 8 (Muthén & Muthén, CA, USA) [25]. Dichotomous variables were recommended for analysis to facilitate the interpretability of the findings from the LCA. All variables used in this analysis from the original Health Examination in School were dichotomous, so there was no need for re-coding the variables. To determine the appropriate model, a step-wise approach was performed, increasing the number of latent classes, until no improvement was observed. Combinations of model fit indexes, such as Akaike information criteria (AIC), Bayesian information criteria (BIC), the Lo-Mendell-Rubine likelihood ratio test (LMR-LRT), and entropy, were compared. For AIC and BIC, smaller values indicate better model fitness, while changes between the models with k and k-1 classes higher than 0.05 means no significant improvement in LMR-LRT [26]. For entropy, which is measured with a range of 0 to 1, higher values are preferred [27].

## 3. Results

### 3.1. Classes Selection

Table 1 provides the LCA fit statistics for the two- to five-class models. According to the statistical values, the most adequate overall model was four classes for both male and female adolescents. The four-class model for male adolescents showed the lowest AIC, a significant LMR-LRT *p*-value, and the highest entropy, while the lowest BIC was found in the three-class model. For female adolescents, the four-class model showed the lowest AIC, a statistically significant LMR-LRT *p*-value, and the highest entropy, although the BIC was not the lowest.

### 3.2. Classes among Male Adolescents

**Figure 1:**
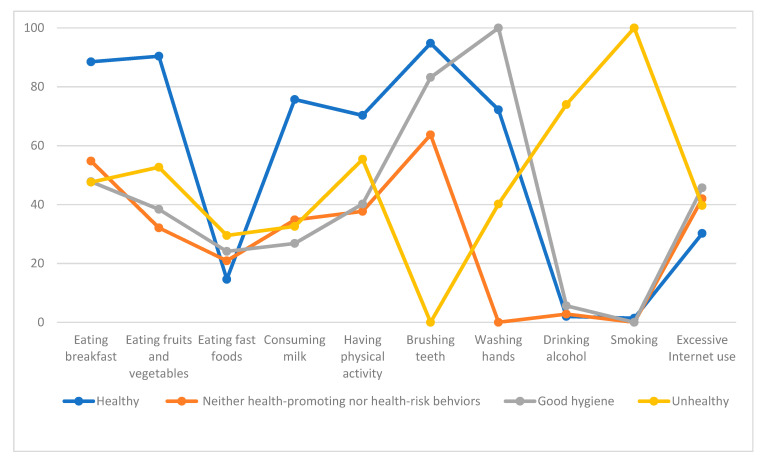
Health-related behavioral characteristics of each class among male adolescents.

A graphical representation of the four classes among male adolescents is shown in Figure 1. The x-axis shows the health-related behaviors included in this analysis, and the y-axis represents the percentages of having specific health-related behaviors within each class. The percentages are within-class individual characteristics different from those in other classes. Table 2 presents the descriptive statistics of health-related behaviors for each class. Group 1, the largest group, was the healthy group. Male adolescents in this group had breakfast regularly, ate fruits and vegetables or consumed milk or dairy products every day, engaged in vigorous physical activity at least three times per week, brushed their teeth more than twice a day, and washed their hands. About one-fourth were clustered as group 2. These adolescent males were less likely to eat fruits and vegetables, consume milk or dairy products, and wash their hands; however, they tended not to drink alcohol or smoke. Group 3, the attentive only to hygiene group, had a high proportion of personal hygiene, both brushing teeth and washing hands, but lower levels of eating fruits and vegetables, consuming milk or dairy products, drinking alcohol, and smoking. The smallest group, group 4, consisted of unhealthy male adolescents who reported a higher proportion of drinking alcohol and smoking. The proportion of those who engaged in excessive Internet use ranged from 30.2 to 45.7.

### 3.3. Classes among Female Adolescents

**Figure 2:**
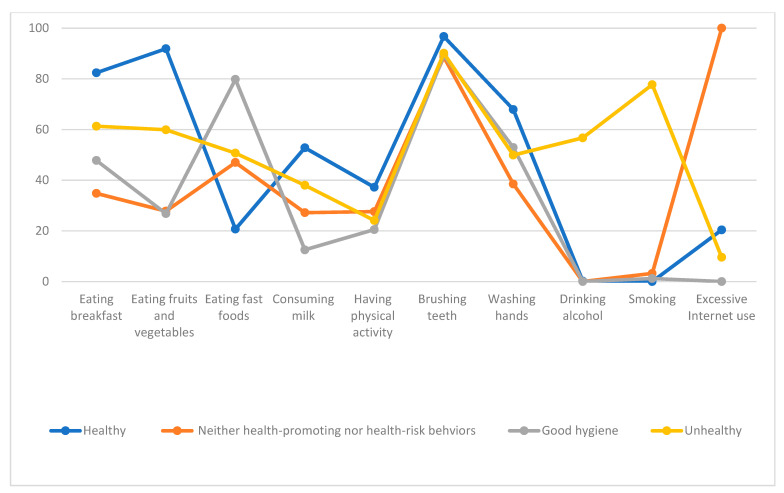
Health-related behavioral characteristics of each class among female adolescents.

Figure 2 provides a graphical representation of the four classes among female adolescents and Table 3 provides the descriptive statistics of each class. As with the male adolescents, Group 1 was the healthy group, which was also the largest group. It consisted of female adolescents who had a higher probability of eating breakfast regularly, eating fruits and vegetables every day, and having good personal hygiene, and with a lower probability of drinking alcohol and smoking. About 17% of female adolescents were classified as group 2. This group was more likely to use the Internet excessively, while being less likely to have good eating and exercise habits, drink alcohol, and smoke. Group 3 included female adolescents who were only attentive to personal hygiene, reporting a higher level of brushing their teeth and washing their hands. They also reported lower levels of eating fruits and vegetables, consuming milk or dairy products, and engaging in vigorous physical activity. Group 4 was the smallest group, which included female adolescents with a higher probability of drinking and smoking, and a lower probability of good eating habits and vigorous physical activity.

## 4. Discussion

The aim of the present study was to identify the latent classes of Korean adolescents by gender based on their health-related behaviors. Using LCA, four latent classes were identified regardless of gender: (1) a group with healthy behaviors, (2) a group without either health-promoting or health-risk behaviors, (3) a group with only good hygiene behaviors, and (4) a group with unhealthy behaviors. The patterns of grouping were relatively consistent across genders. The majority of both male and female adolescents were classified into the healthy behaviors group (50.5% and 51.4% respectively). The lowest frequencies for both male and female adolescents were for the unhealthy behaviors group. However, a higher proportion of boys comprised the unhealthy group (6.6% vs. 3.7%).

Depending on gender, the important behaviors for grouping were found to be different. For boys, eating breakfast, eating fruits and vegetables, consuming milk, brushing teeth, washing hands, drinking alcohol, and smoking were important. However, for girls, eating breakfast, eating fruits and vegetables, eating fast food, drinking alcohol, smoking, and excessive Internet use were important. Interestingly, at least about 40.0% of boys reported that they engage in vigorous physical activity three times or more per week, while for girls, the highest proportion of engaging in such activity was 37.2%. This is consistent with previous studies that found that boys were more physically active [8,9]. In addition, both boys and girls who exhibited unhealthy behaviors were more likely to have had an experience of drinking alcohol and smoking. However, more boys were exposed to drinking alcohol and smoking than were girls, which was consistent with findings from other studies [13,14,15].

The finding that about half of each gender group were classified as adolescents who had healthy behaviors is consistent with the findings from the study of adolescents in Australia [23,28]. However, in the United States, the highest proportion of male adolescents were classified as having unhealthy behaviors, while the mixed group had the highest proportion of female adolescents, based on data from Add Health in 1994 (Wave I) and 2008 (Wave IV) [24]. Compared to the study in the United States, Korean adolescents in the present study were more likely to have healthy eating habits, and less likely to smoke and consume alcohol. Although a Westernized diet in Korea has steadily been increasing, many Korean adolescents prefer the traditional Korean diet, which is considered healthy. In addition, diverse types of tobacco products or drugs are not available to adolescents in Korea. Another factor is the difference in the timing of the two studies. These factors could contribute to the differences between the findings of this study and the U.S. study. The proportion of the unhealthy group, which was the smallest group in this study, is comparable to studies in the United States and Australia. In Xiao et al.’s study, about 7% of U.S. adolescents were classified into the low health-promoting behaviors group, using data from the 2017 Youth Risk Behavior Survey [22]. In the study of female Australian adolescents, the smallest class was approximately 5%, characterized by excessive screen time, low physical activity, and low vegetable intake [28]. Regardless of countries, adolescents with unhealthy behaviors consisted of the smallest proportion, although the behaviors that characterize the cluster differ.

Although the healthy group was the largest group both in male and female adolescents, boys were more likely than girls in the healthy group to be physically active. The higher probability of girls not engaging in vigorous physical activity was found in the other groups as well. In general, studies show boys tended to exercise more than girls [8,29,30]. However, healthy groups from other countries were more likely to engage in physical activity regardless of gender. In the studies from the United States and Australia, adolescents were asked to answer whether they engaged in moderate to vigorous activities or whether they participated in physical activities [23,24,28]. However, in the present study, only vigorous physical activity was assessed. Female adolescents might focus more on other types of physical activity, like Pilates, yoga, or stretches. Previous studies concluded that the amount of exercise, regardless of the intensity, was associated with improvement in one’s health status, although high intensity exercise was considered critical for health related to obesity, blood lipid levels, and psychological well-being [31,32]. Thus, it would be necessary to explore the types and amount of exercise that female adolescents engage in to identify the reasons why they are less likely to engage in vigorous physical activity. Such information would have utility for planning appropriate approaches to encourage female adolescents to be physically active.

With regard to the difference in gender in the unhealthy group, for boys the probability of drinking alcohol was about 75% and that of smoking was 100%. Other studies have found the same trend wherein alcohol consumption and smoking was higher among boys than girls [33,34]. In the present study, those behaviors were not important for clustering among girls. This finding was consistent with the Australian study identifying that alcohol consumption and smoking were not found to be essential factors for clustering among female adolescents [28]. Alcohol consumption and tobacco use are well-known as influential factors for various health problems, including cardiovascular diseases, cancers, liver disease, and lung disease [35,36,37]. In addition, many studies have found that these behaviors were associated with delinquent behaviors [38,39], and that they could persist into adulthood [3,7]. Therefore, appropriate interventions to address male adolescents’ alcohol consumption and tobacco use are necessary.

More than 40% of male and female adolescents were included in the other two groups: the group without either health-promoting or health-risk behaviors, and the group with only good hygiene behaviors. Adolescents whose health-related behaviors are consistent with these two classifications should be given special attention, because there is a chance that they could improve their health-related behaviors with proper guidance. To encourage health-promoting behaviors, it would be necessary to first identify the underlying reasons that impede healthy eating or exercise behaviors. With this knowledge, exploration of motivating factors, what resources they need, and how to provide those resources would be helpful.

Interestingly, 100% of female adolescents without either health-promoting or health-risk behaviors responded that they spent at least two hours daily using the Internet. Among male adolescents, Internet use was not an essential behavior. For female Australian adolescents, the amount of screen time was also one of the influential behaviors to cluster [28]. Generally, Internet use is considered sedentary behavior, which is associated with lower physical activity [40,41]. Increased sedentary activity and decreased physical activity are modifiable influential factors that contribute to obesity and the development of various diseases, such as cardiovascular disease [5,41]. In addition to inappropriate posture when using the Internet, various musculoskeletal symptoms like headache, neck pain, and wrist pain can result from excessive Internet use [42,43,44]. Thus, before various problems emerge, preventative measures should be considered. Because this group spends more time using the Internet, campaigns or catchphrases provided through the Internet would be more accessible for them.

The group with only good hygiene behaviors had a lower proportion of healthy behaviors; however, good hygiene could prevent various contagious diseases, including the spread of COVID-19 [45,46]. Indeed, brushing teeth regularly could be helpful for dental health [47]. By exploring the reason why adolescents in this group are more concerned with personal hygiene, we could identify the difference between this group and others, and the factors influencing performing good hygiene behaviors. Based on the findings, strategies for the other groups could be developed to motivate them to have good hygiene practices.

There are several limitations to this study. First, this study involved secondary data analysis; therefore, other variables that are potentially associated with health-related behaviors were not able to be considered. Further, the questionnaires of the original data were measured as single-items, and the errors in the measurement was not considered. Thus, the reliability of the measurements is required for further studies. Furthermore, because this was the descriptive study, the findings from this study could not be predictive or causal. Next, the variables included in this study were assessed by self-report, which could be affected by response bias and social desirability. Finally, the findings of the present study, including the models of classes, are limited in the generalizability to other adolescent populations. The presented model is provisional and it could be contrasted in independent samples.

## 5. Conclusions

In this study, we identified the clustering of health-related behaviors among Korean adolescents by gender, using LCA. Four classes were determined across genders: a group with healthy behaviors, without either health-promoting or health-risk behaviors, with good hygiene only, and with unhealthy behaviors. The largest group was the healthy behaviors group, while the smallest was the unhealthy behaviors group. Smoking and drinking alcohol for male adolescents and Internet use for female adolescents were also the most significant behaviors for patterning among adolescents. To improve adolescents’ health-related behaviors, tailored interventions that consider the behavioral features of each group are necessary.

## Figures and Tables

**Table 1:** Model fit statistics for 2- to 5-class models (n = 1807).

Model Fit Indexes	Number of Classes			
	2	3	4	5
Male (n = 1129)				
AIC	11,726.242	11,542.323	11,535.073	11,520.681
BIC	11,831.759	11,703.116	11,751.133	11,792.012
LMR-LRT	0	0	0.0078	0.2272
Entropy	0.531	0.715	0.771	0.729
Female (n = 678)				
AIC	6488.399	6441.904	6428.342	6430.781
BIC	6583.177	6586.327	6622.411	6674.496
LMR-LRT	0	0.007	0.0443	0.2822
Entropy	0.493	0.666	0.745	0.741

**Table 2:** Proportions of health-related behaviors of male adolescents by the classes (%).

Health-Related Behaviors	Class 1 (n = 568)	Class 2 (n = 280)	Class 3 (n = 201)	Class 4 (n = 80)
*Eating habits*				
Eating breakfast everyday	88.5	54.8	47.8	47.6
Eating fruits and vegetables everyday	90.4	32.1	38.4	52.7
Eating fast foods everyday	14.6	20.9	24.1	29.5
Consuming milk and dairy product everyday	75.7	34.8	26.8	32.6
*Physical activity*				
Having vigorous physical activity 3 times or more per week	70.3	37.7	40.2	55.4
*Personal hygiene*				
Brushing teeth more than twice a day	94.8	63.7	83.2	88..8
Washing hands before eating food or after going out	72.2	0.0	100.0	40.2
*Drinking alcohol*				
Having any experience of drinking alcohol in the past 30 days	2.0	2.8	5.6	74.0
*Smoking*				
Having any experience of smoking in the past 30 days	1.4	0.0	0.0	100.0
*Internet use*				
Using internet, including internet games, two hours and more per day.	30.2	42.0	45.7	39.7

**Table 3:** Proportions of health-related behaviors of female adolescents by the classes (%).

Health-Related Behaviors	Class 1 (n = 347)	Class 2 (n = 113)	Class 3 (n = 189)	Class 4 (n = 25)
*Eating habits*				
Eating breakfast everyday	82.4	34.8	47.8	61.3
Eating fruits and vegetables everyday	91.9	27.8	26.8	59.9
Eating fast foods everyday	20.7	47.0	79.8	50.7
Consuming milk and dairy product everyday	52.8	27.2	12.5	38.0
*Physical activity*				
Having vigorous physical activity 3 times or more per week	37.2	27.6	20.5	24.1
*Personal hygiene*				
Brushing teeth more than twice a day	96.7	88.6	89.3	90.1
Washing hands before eating food or after going out	67.9	38.5	52.9	49.9
*Drinking alcohol*				
Having any experience of drinking alcohol in the past 30 days	0.2	0.0	0.0	56.7
*Smoking*				
Having any experience of smoking in the past 30 days	0.0	3.2	1.2	77.7
*Internet use*				
Using internet, including internet games, two hours and more per day.	20.4	100.0	0.0	9.6

## Data Availability

Publicly available datasets were analyzed in this study. This data can be found at [http://www.schoolhealth.kr/web/srs/selectPrivacyAgree.do?sMenuId=0100008800] (accessed on 4 February 2021).
